# Parasitological and Bacteriological Evaluation of Common Raw Fruits Collected from Two Major Markets in Iwo, Osun State, South-West Nigeria

**DOI:** 10.1155/2023/5524499

**Published:** 2023-05-18

**Authors:** Oladipo O. Oladosu, David O. Olatunde, Adewale O. Olatayo, Bukola Atobatele

**Affiliations:** ^1^Pure and Applied Biology Programme, Bowen University Iwo, Osun State, Nigeria; ^2^Microbiology Programme, Bowen University Iwo, Osun State, Nigeria

## Abstract

**Background:**

Fruits are a vital part of human nutrition because they contain the growth factors required to maintain normal health. Fruits are also known to harbour a wide range of parasites and bacteria. Consumption of unwashed raw fruits can lead to foodborne pathogens. This study was carried out to examine the presence of parasites and bacteria on fruits sold at two major markets in Iwo, Osun state, South-West Nigeria.

**Methods:**

Twelve different fresh fruits and seven different fresh fruits were purchased from different vendors from “Odo-ori” and “Adeeke” markets, respectively. The samples were transported to the microbiology laboratory of Bowen University, Iwo, Osun state for bacteriological and parasitological analysis. The parasites were concentrated by sedimentation and were examined using a light microscope, while for the microbial analysis, culturing and biochemical tests were also carried out on all the samples.

**Results:**

The parasites discovered include *Fasciola hepatica* eggs, *Ascaris lumbricoides* and *Strongyloides stercoralis* larvae, hookworm larvae, and *Taenia* and *Trichuris trichiura* eggs. *Ascaris lumbricoides* was most frequently detected (40.0%). Bacteria isolated from the sampled fruits include *Klebsiella oxytoca*, *Klebsiella pneumoniae*, *Enterobacter aerogenes*, *Escherichia coli*, *Kluyvera ascorbata*, *Proteus mirabilis*, *Staphylococcus intermedius*, *Staphylococcus* sp., *Micrococcus sedentarius*, *Corynebacterium diphtheriae*, and *Streptococcus zooepidemicus*.

**Conclusion:**

The presence of parasites and bacteria on the fruits observed indicates that public health diseases may arise from their consumption. Awareness and education among farmers, vendors, and consumers about the importance of personal and food hygiene through proper washing or disinfection of fruits could reduce the risk of parasites and bacterial fruit contamination.

## 1. Introduction

Fruits are fundamental sources of essential vitamins, and they boost host immunity and contain antioxidants and fibres [1]. Nearly every fruit, in some form or another, has beneficial properties. Fruits can aid the treatment of some diseases, such as scurvy, night blindness, asthma, fever, anaemia, and ulcers, and their daily consumption plays a key role in the prevention of certain nutritional deficiencies, cardiovascular diseases, and stroke [2].

Fruits have many health and economic advantages, but there is also a lot of worry that they could become contaminated with human pathogens after being eaten fresh or lightly cooked [3]. They sometimes act as a medium for the spread of infectious diseases, causing foodborne diseases of public health importance, resulting in morbidity and mortality [4]. Fruits and vegetables can transmit infectious organisms and pose a great health threat [5]. Fruits that have been damaged during growth, harvesting, or handling, such as those that have splits, wounds, or punctures, can become infected by pathogenic organisms [6].

Foods can become contaminated widely as a result of factors like a lack of latrines and poor sewage disposal facilities, which are known to help spread parasitic infection states. Inadequate hygienic procedures used during the planting, harvesting, packing, transportation, and storage of fruits and vegetables can easily lead to contamination [7]. Research on diverse fruit and vegetable samples have indicated that *Ascaris lumbricoides*, *Cryptosporidium* spp., *Entamoeba histolytica*, *Enterobius vermicularis*, *Fasciola* spp., *Giardia lamblia*, hookworms, *Hymenolepis* spp., *Taenia* spp., *Trichuris trichiura*, *Cyclospora* spp., and *Toxocara* spp. infect humans who consume contaminated fruits and vegetables that have not been adequately cooked or washed [8–10].

Increased risks of infection with parasitic organisms have been reported in different areas, including Nigeria, where untreated wastewater is being used for the irrigation of fruits and vegetables [11, 12]. Animal excrement is used as manure, and the poor sanitary habit of eating fruits without washing contributes to the spread of these diseases. Most fruit sellers use poor quality water to wash the fruits before sale to consumers, thereby exposing them to the risk of both microbial and parasitic infections [13]. Information about the degree of parasitic and microbial infection of fruits sold in many markets in Osun state, including Iwo town where this study was conducted, is lacking. As a result, this study was conducted to determine the occurrence of parasitic and bacterial infestations of common fruits in the two main markets in Iwo town.

## 2. Materials and Methods

### 2.1. Study Area and Design

**Figure 1:**
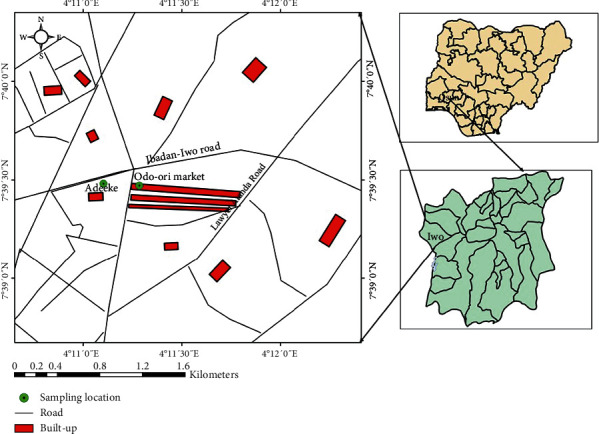
Map of location of Adeeke and Odo-ori markets in Iwo, Osun state, Nigeria.

The study was conducted at two major markets in Iwo town in the Iwo local government area of Osun state, South-West Nigeria. The two markets (Odo-ori and Adeeke) are the largest markets in Iwo town, and people from different surrounding states and towns come to sell and buy goods.  Figure 1 shows the map of the Adeeke and Odo-ori markets between the latitudes 7°39′0^″^N and 7°40′0^″^N and longitudes 4°11′0^″^E and 4°12′0^″^E. Goods sold in the market are from farmers in the town and nearby states and communities. The major market day occurs every five-day interval, and this happens at different days for the two markets where the sampling was done. The study was a cross-sectional sampling done in June and July of 2021.

### 2.2. Sample Collection

The fruit samples were obtained separately from the two markets that were visited on different market days and placed in sterile polythene bags that were properly labeled with a distinctive number and sampling date. All available fruits were purchased at the markets during the sample collection period. One sample of each fruit was used for bacteriological and parasitological analysis. A total of twelve (12) different fresh fruits that include watermelon (*Citrullus lanatus*), tomato (*Solanum lycopersicum*), tangerine (*Citrus reticulata*), banana (*Musa paradisicum*), mango (*Mangifera indica*), orange (*Citrus aurantium*), cucumber (*Cucumis sativus*), garden egg (*Solanum melongena*), apple (*Malus domestica*), pear (*Persea americana*), pineapple (*Ananas comosus*), and pumpkin (*Cucurbita moschata*) were sampled from the first market known as “Odo-ori,” and a total of seven (7) fruits including garden egg (*Solanum melongena*), orange (*Citrus aurantium*), banana (*Musa paradisicum*), apple (*Malus domestica*), pineapple (*Ananas comosus*), watermelon (*Citrullus lanatus*), and cucumber (*Cucumis sativus*) were collected from the second market known as “Adeeke.” The samples were taken to Bowen University's microbiology laboratory (Iwo, Osun state) for bacteriological and parasitological analysis. Every fruit obtained from the two marketplaces (Adeeke and Odo-ori) was tested for parasites and bacteria.

### 2.3. Parasitological Analysis

The market samples were immediately transferred to the laboratory, where 10 g of each fruit was weighed and placed in sterile beakers with a saline solution (0.9% NaCl) and then washed. New gloves were used for each fruit analysed. Fruits, such as banana, tangerines, and oranges, were cut through the peel and fruit, and 10 g of each fruit was used for the analysis. The parasitological analysis was conducted using the centrifugal sedimentation technique as described in [14]. Briefly, in order to remove eggs, larvae, and cysts from the fruits, the procedure involved the fruits being soaked into a sterile saline solution and agitating them five times in the span of 30 minutes. The suspension was filtered through a sterile, clean fine mesh gauze to remove larger debris. The filtrate was centrifuged at 5000 g for 5 minutes, the supernatant was removed after centrifugation without shaking, and the sediment was meticulously examined for parasite ova, larvae, and cysts using a light microscope with an objective lens of 10 and 40. Parasite ova, larvae, and cysts were compared to and identified using known features described elsewhere [14].

### 2.4. Microbial Analysis

Additional fruit samples weighing 10 g each were washed in 100 ml of sterile distilled water and added to new sterile test tubes containing 9 ml of sterile distilled water in which 1 ml of the sample water was used for serial dilution to 10^−2^ using sterile syringes. Finally, 1 ml of the 10^−2^ for every sample was pipetted into sterile petri dishes.

### 2.5. Culturing Technique

The media used in this study were MacConkey agar (selective and differential) and nutrient agar (a general-purpose medium), which were dispensed into sterile petri dishes and allowed to cool and solidify. The sterile molten agars were poured into the appropriate petri dish and allowed to solidify. The petri dishes were inverted into the incubator. The cultured media were incubated at a temperature of 37°C for 24–48 hours.

The petri dishes were examined for primary identification based on colonial characteristics after incubation. To identify the bacteria isolated from the fruits, different morphologic and biochemical tests were performed on the pure cultures of the bacteria [15].

### 2.6. Bacterial Isolates' Morphology and Biochemical Characterization

The following cellular morphology and biochemical characteristics were carried out on the bacteria to confirm their identity.

### 2.7. Gram Staining

On clean, grease-free slides marked with the code for each isolate, a smear of 18-hour-old pure culture was made using a sterile wire loop. The slides' film was air dried before being gently heated and fixed. After flooding the fixed smear for 30 seconds with a crystal violet primary stain, iodine was then added for 60 seconds to create a crystal violet-iodine complex. The stained smear was immediately flooded with water after being decolored with 70% ethanol. The smear was rinsed with and the blot dried after being counter stained with safranin dye for 30 seconds. The slides were then viewed under a microscope with an oil immersion lens. Gram-positive cells are purple since they are not decolorized with ethanol and retain the purple color. Gram-negative cells are red. This is because ethanol treatment removes the crystal violet-iodine complex [15].

### 2.8. Biochemical Test

#### 2.8.1. Methyl-Red Test

A sterile test tube was used to inoculate the isolate into glucose phosphate peptone water, which was then incubated at 37°C for 48 hours. Five drops of a methyl-red indicator solution were then added, mixed, and immediately inspected. Yellow coloration denotes a negative reaction, while red coloration denotes a positive methyl-red-positive test.

#### 2.8.2. Voges-Proskauer Test

As instructed for the methyl-red test, the isolate was also cultured in glucose phosphate peptone water. The O'Meara reagent, which consists of 40 g of KOH and 0.3 g of creatinine dissolved in 100 ml of distilled water, was added after the incubation period. After being placed in a water bath at 37°C for 4 hours, the test tubes were removed, mixed, and read within 30 minutes. The test tubes were periodically shaken. A yellow coloration indicates a negative reaction to the Voges-Proskauer test, while an eosin-pink coloration indicates a positive result.

#### 2.8.3. Citrate Utilization Test

Simmons citrate agar was prepared in a beaker, and it was heated on a hot plate for 15 mins, after it was measured inside the test tubes and autoclaved. After solidification, the test tube was inoculated with each of the organism broth cultured in sterilized bottles, respectively. This was incubated at 37°C for 2 days, and then, a citrate-positive colour change from green to blue was observed. There is no change of color in the negative test.

#### 2.8.4. Indole Test

The indole medium was prepared by dissolving 10 g of tryptone water and 5 g of sodium chloride in 1000 ml of distilled water. Clean test tubes were filled with seven milliliters of the medium, which was then sterilized at 121°C for 15 minutes. The test organisms were then injected into the medium, which was then incubated for 48 hours at 35°C. Three drops of Kovac's reagent were added and gently shaken after 48 hours. Positive reaction is indicated by the alcohol layer turning red [16].

#### 2.8.5. Sugar Fermentation Test

The medium used was 1.0% peptone water, 0.1% sodium chloride (NaCl), and a 0.01% phenol red indicator. Sugar liquid was prepared by adding 1% of the particular sugar (glucose, sucrose, and lactose). Seven milliliters of sugar broth were dispensed into the test tubes. Durham tubes were placed inverted in each test tube leaving no air space in the Durham tubes. The medium in the test tube was then sterilized at 121°C for 15 minutes in an autoclave. The medium was then inoculated with the test organism and incubated at 35°C for 72 hours. Fermentation of the sugar present is indicated by a change in the color of the medium from red to yellow.

### 2.9. Data Analysis

Data were entered into Microsoft Excel and analyzed using the SPSS 25.0 software (SPSS Inc., Chicago, IL, USA). *p* values were calculated using the chi-square test appropriately. A *p* value < 0.05 was considered statistically significant.

## 3. Results

A total of twelve different fresh fruits that were available at the time of sample collection were bought from the first market known as “Odo-ori.” In the second market, known as “Adeeke,” a total of seven different fresh fruits available at the time of sample collection were purchased. The common and botanical names of the purchased fruits from the two markets where the samples were collected are as shown in Table 1. The botanical identification of the fruits were done at the Plant Biology Unit of the Pure and Applied Biology Programme, Bowen University, Iwo by a plant taxonomist.

The total number of different parasites detected in the fruits collected from the two markets was six. A total of seven (33.3%) and three (42.9%) parasites were detected in the first (Odo-ori) and second (Adeeke) markets, respectively (Table 2). The majority of the parasites seen was *Ascaris lumbricoides*, 4 (40.0%).

Comparison of the total plate count of sampled fruits between the two markets indicates that there was no significant difference (*p* = 0.680) in the plate count of the sampled fruits between the Odo-ori and Adeeke markets (Table 3).

Table 4 lists the different bacterial isolates discovered in the examined fruits along with their macroscopic, microscopic, and biochemical descriptions.

The biochemical test results of different bacterial isolates observed in the first market, “Odo-ori,” and the second market, “Adeeke,” revealed a total of twenty-one (21) isolates from the Odo-ori market (Table 5) and eleven (11) isolates from the Adeeke market (Table 6). The association between markets and bacteria isolates seen in fruits based on biochemical tests showed the following results. The citrate test result showed a statistical significance result (*p* = 0.005), with almost all sampled fruits from the Odo-ori market displaying positive results with bacterial infection (95.2%). In the Adeeke market, only 45.5% of fruits were infected with bacterial isolates. The Gram staining test, indole test, methyl-red test, Voges-Proskauer test, lactose gas test, and sucrose gas test comparing the two markets were all statistically significant (Table 7).

## 4. Discussion

Fruits are an edible and essential part of the human diet. They provide the growth factors required to maintain normal health. The detection of parasites and bacteria in this study is indicative of faecal contamination of fruits by humans as well as animals. Parasites and bacteria are widely distributed in many tropical countries including Nigeria owing to favourable climatic conditions for their existence and spreading, as well as the unhygienic states that enable faecal contamination of water, food stuffs, and soil [17]. Because the farmers' and vendors' hygiene are unknown, purchasing ready-to-eat fruit directly from vendors may potentially increase the risk of consuming a foodborne pathogen.

This study was aimed at evaluating parasitic and bacteriological infestations of some common fruits sold in two major markets in Iwo town, Nigeria. The total number of different parasites detected in this study was six. This study is comparable to some studies that have the similar level of parasites [8]. This is, however, in contrast to some studies that detected more [8, 13, 18] and less [19] types of parasites in the fruits sampled. A study of freshly harvested and “ready-to-eat” vegetables reported several zoonotic parasites [11]. Factors such as environmental conditions, the kind of sample, the sample technique employed, the socioeconomic status, and the methods of detection may be the reason for the variation in the types of different parasites reported in this study. Untreated wastewater has been reported to be a major source of parasitic contamination of fruits and vegetables [20, 21]. Using human faeces as fertilizer could also be a source of contamination for fruits and vegetables [22, 23]. Possible contamination of vegetables via animal dung has been reported as some of the parasites reported, such as *Toxocara* spp., *Ancylostomatidae*-*Strongylidae*, Coccidian oocysts (*Toxoplasma* spp.), and *Echinococcus* spp., were zoonotic parasites [7]. Handling of fruits during or after harvest, including handling from the farm to the market where it will be purchased, may lead to contamination of the fruits.

Out of the parasites detected in this study, *Ascaris lumbricoides* was more prevalent than any other parasite encountered. This is in agreement with findings reported elsewhere [20, 24, 25]. *Strongyloides stercoralis* was, however, reported as the most frequently encountered parasite in some similar studies [17, 23]. Other studies reported *Cryptosporidium* spp., *Entamoeba histolytica*/*dispar*, and eggs of *Trichostrongylus* as the predominant parasites detected [12, 13]. The widespread distribution of *A. lumbricoides*, the large number of eggs produced by the fertile female parasite, which facilitates the parasite's global distribution, and the hardiness and resilience of the eggs, which enable them to survive unfavourable conditions, could all be contributing factors to the parasite's dominance. *Ascaris* eggs can survive without oxygen for two years at lower temperatures and can withstand desiccation for two weeks [26].

The presence of some parasites isolated in this study, such as *Ascaris lumbricoides*, *Strongyloides* sp., *Trichuris trichiura*, and hookworm, suggests that the contamination may be mainly through faecal origin. This may be due to the poor sanitary conditions and hygienic habits of the farmers or vendors [27]. Insects, such as the housefly (*Musca domestica*), have transmitted intestinal parasites in infected human faeces [28].

The fruits examined in this study were also contaminated by various bacteria apart from the parasites reported. This agrees with the study reported earlier that discovered the presence of both parasites and microbes in fruits [5]. The load of microorganisms observed in this study may be linked to factors such as the unsanitary condition of where the fruits have been purchased or transportation systems of farms. Vendors, inappropriate handling of fruits, and mixing of contaminated fruits with good ones during storage or packing may lead to the spread of bacteria from contaminated fruits to the good ones. Environmental sources, such as faeces or untreated sewage, and microorganisms present in the soil or water can be the source of contamination for fruits [29].

The different bacteria isolated in this study showed that fruits could act as a medium for the spread of pathogenic microbes. The organisms isolated in this study were similar to those in studies reported earlier [25]. *Escherichia coli* (coliforms) and *Proteus mirabilis* (enteric bacteria) isolation from sampled fruit suggested the possibility of faecal contamination or use of human faeces as manure [30]. Inappropriate storage of fruits in a favourable condition for bacterial growth could increase the load of bacteria in fruits. When fruits are stored at inappropriate temperatures, they tend to attain temperatures that are suitable for microbial growth pathogens to cause disease when ingested [31].

Isolation of *Staphylococcus* spp. in this study may be linked to vendor body contact with the sampled fruits because the organism is a normal flora of the nasal passage, hands, and skin of healthy individuals, and it has been reported to have the highest occurrence in fruits and foods [32]. The occurrence of *Staphylococcus aureus* in food is a sign of environmental and human contamination [31]. The pathogens that were isolated, *Escherichia coli*, *Staphylococcus* sp., and *Klebsiella* spp., are established opportunistic pathogens that are important for public health.

The appearance of fruits may not be a good indicator of their microbial quality before consumption. Before eating any fruit, it should be thoroughly washed. Fruits should be washed by both vendors and consumers, and decontaminants such as vinegar should be included in the wash water where possible. There is a need to properly educate vendors to observe strict hygiene so as not to serve as a source of contamination to fruits. It is also necessary that the sources of irrigation or wells be monitored to limit the introduction of pathogenic bacteria and parasites. Before applying manure as a fertilizer, it must be properly treated. Manure used as fertilizer should be treated by either composting or aging to eliminate pathogenic microorganisms, and farmers should be educated on the importance of allowing enough time between the final application of manure and harvest [1].

## 5. Conclusion

This study has revealed various parasites and bacteria in fruits that can lead to public health crises that could result in infections with joined symptoms showing the pathogens concerned. It is also of importance that farmers and vendors should be properly educated on the hygiene procedure in farms and markets to reduce the incidence of parasites and microorganisms likely to be seen in fruits. The consumers need awareness on the usefulness of proper washing and disinfecting of fruits before consumption in order to prevent parasitic and bacterial illnesses.

## Figures and Tables

**Table 1:** List of raw fruits collected at the two major markets in Iwo town.

S/N	Common name	Botanical name
*Odo-ori market*		
1	Watermelon	*Citrullus lanatus*
2	Tomato	*Solanum lycopersicum*
3	Tangerine	*Citrus reticulata*
4	Banana	*Musa paradisicum*
5	Mango	*Mangifera indica*
6	Orange	*Citrus aurantium*
7	Cucumber	*Cucumis sativus*
8	Garden egg	*Solanum melongena*
9	Apple	*Malus domestica*
10	Pear	*Persea americana*
11	Pineapple	*Ananas comosus*
12	Pumpkin	*Cucurbita moschata*

*Adeeke market*		
1	Garden egg	*Solanum melongena*
2	Orange	*Citrus aurantium*
3	Banana	*Musa paradisicum*
4	Apple	*Malus domestica*
5	Pineapple	*Ananas comosus*
6	Watermelon	*Citrullus lanatus*
7	Cucumber	*Cucumis sativus*

**Table 2:** Frequency of parasite found in fruits in the two markets.

Parasites	Markets		Total *N* (%)
	Odo-ori *N* (%)	Adeeke *N* (%)	
Hookworm	1 (14.3)	0 (0.0)	1 (10.0)
*Ascaris* spp.	4 (57.2)	0 (0.0)	4 (40.0)
*Fasciola* sp.	1 (14.3)	0 (0.0)	1 (10.0)
*Strongyloides* spp.	1 (14.3)	1 (33.3)	2 (20.0)
*Taenia* sp.	0 (0.0)	1 (33.3)	1 (10.0)
*Trichuris* sp.	0 (0.0)	1 (33.3)	1 (10.0)
Total parasite	7 (100.0)	3 (100.0)	10 (100.0)

**Table 3:** Differences in plate count in fruits between the two markets.

Markets	*N*	Mean	Standard deviation	*t*-calc.	*p* value
Odo-ori	12	24.00	23.26	-0.420	0.680
Adeeke	7	30.71	47.07		

**Table 4:** Types of organisms isolated in the two markets sampled in Iwo town.

S/N	Gram-positive organisms	Gram-negative organisms
Odo-ori market		
1	*Klebsiella oxytoca*	*Staphylococcus intermedius*
2	*Klebsiella pneumoniae*	*Micrococcus sedentarius*
3	*Enterobacter aerogenes*	*Corynebacterium diphtheriae*
4	*Escherichia coli*	*Proteus mirabilis*
5	*Kluyvera ascorbata*	*Staphylococcus* sp.

Adeeke market		
1	*Klebsiella oxytoca*	*Streptococcus zooepidemicus*
2		*Corynebacterium diphtheriae*
3		*Staphylococcus* sp.

**Table 5:** Biochemical test results of isolates in Odo-ori market.

Code	Gram	CRT	INDO	MR	VP	LACT	GAS	GLU	GAS	SUC	GAS	Potential org.
1a	-ve	+ve	-ve	-ve	+ve	+ve	+ve	+ve	-ve	-ve	-ve	*Kluyvera ascorbata*
b	+ve	-ve	-ve	-ve	-ve	-ve	-ve	-ve	+ve	+ve	+ve	*Streptococcus uberis*
2a	-ve	+ve	-ve	-ve	+ve	+ve	+ve	+ve	+ve	+ve	+ve	*Escherichia coli*
b	+ve	+ve	-ve	+ve	+ve	+ve	+ve	+ve	+ve	+ve	-ve	*Staphylococcus intermedius*
3a	-ve	+ve	+ve	+ve	+ve	+ve	+ve	+ve	+ve	+ve	+ve	*Klebsiella oxytoca*
b	-ve	+ve	-ve	-ve	-ve	+ve	-ve	-ve	-ve	+ve	-ve	*Escherichia coli*
4	+ve	+ve	-ve	+ve	-ve	+ve	+ve	+ve	+ve	+ve	-ve	*Micrococcus sedentarius*
5	-ve	+ve	-ve	+ve	+ve	+ve	+ve	+ve	+ve	+ve	-ve	*Enterobacter aerogenes*
6a	+ve	+ve	-ve	-ve	+ve	+ve	-ve	+ve	+ve	+ve	-ve	*Staphylococcus intermedius*
b	+ve	+ve	+ve	+ve	+ve	-ve	-ve	+ve	+ve	+ve	-ve	*Corynebacterium diphtheriae*
7a	-ve	+ve	+ve	+ve	-ve	+ve	+ve	+ve	+ve	+ve	+ve	*Escherichia coli*
b	+ve	+ve	+ve	+ve	-ve	+ve	+ve	+ve	+ve	-ve	-ve	*Proteus mirabilis*
8a	-ve	+ve	-ve	+ve	+ve	+ve	-ve	-ve	-ve	+ve	+ve	*Klebsiella pneumoniae*
b	+ve	+ve	-ve	-ve	-ve	+ve	+ve	-ve	-ve	+ve	-ve	*Micrococcus luteus*
9a	+ve	+ve	-ve	+ve	-ve	+ve	-ve	+ve	-ve	+ve	-ve	*Corynebacterium diphtheriae*
b	-ve	+ve	-ve	+ve	-ve	+ve	-ve	+ve	+ve	+ve	+ve	*Kluyvera ascorbata*
10	+ve	+ve	-ve	-ve	-ve	+ve	+ve	+ve	+ve	+ve	-ve	*Corynebacterium diphtheriae*
11a	+ve	+ve	+ve	-ve	-ve	+ve	+ve	+ve	+ve	+ve	-ve	*Corynebacterium diphtheriae*
b	+ve	+ve	-ve	+ve	-ve	-ve	-ve	-ve	-ve	+ve	+ve	*Staphylococcus intermedius*
12a	+ve	+ve	+ve	+ve	-ve	+ve	+ve	+ve	+ve	+ve	-ve	*Staphylococcus* sp.
b	-ve	+ve	+ve	-ve	+ve	+ve	+ve	+ve	+ve	+ve	-ve	*Klebsiella oxytoca*

**Table 6:** Biochemical test results of isolates in Adeeke market.

Code	Gram	CRT	INDO	MR	VP	LACT	GAS	GLU	GAS	SUC	GAS	Potential org.
1	+ve	-ve	-ve	+ve	-ve	+ve	+ve	+ve	+ve	+ve	+ve	*Streptococcus zooepidemicus*
2	+ve	+ve	-ve	+ve	-ve	+ve	+ve	+ve	+ve	+ve	+ve	*Corynebacterium diphtheriae*
3A	+ve	+ve	-ve	+ve	-ve	+ve	+ve	+ve	+ve	-ve	-ve	*Corynebacterium diphtheriae*
B	+ve	+ve	-ve	+ve	-ve	+ve	+ve	+ve	+ve	+ve	+ve	*Corynebacterium diphtheriae*
4A	-ve	+ve	-ve	+ve	-ve	+ve	+ve	+ve	+ve	+ve	+ve	*Klebsiella oxytoca*
B	+ve	+ve	-ve	+ve	-ve	+ve	+ve	+ve	+ve	+ve	+ve	*Staphylococcus* sp.
5A	+ve	-ve	-ve	+ve	-ve	-ve	-ve	+ve	+ve	+ve	+ve	*Corynebacterium diphtheriae*
B	+ve	-ve	-ve	+ve	-ve	+ve	+ve	+ve	+ve	+ve	+ve	*Corynebacterium diphtheriae*
6	+ve	-ve	-ve	-ve	-ve	+ve	+ve	+ve	+ve	+ve	+ve	*Streptococcus zooepidemicus*
7A	+ve	-ve	-ve	+ve	-ve	+ve	+ve	+ve	+ve	+ve	+ve	*Corynebacterium diphtheriae*
B	+ve	-ve	-ve	+ve	+ve	+ve	+ve	+ve	+ve	+ve	+ve	*Corynebacterium diphtheriae*

Key: -ve: negative; +ve: positive; Code: organism code; Gram: Gram staining; IND: indole; MR: methyl red; VP: Voges-Proskauer test; CRT: citrate test; LAC: lactose test; GLU: glucose test; SUC: sucrose test.

**Table 7:** Association between markets and bacteria in fruits based on biochemical tests.

Tests	Markets	Positive (%)	Negative (%)	Total	*X* ^2^-calc.	*p* value
Gram staining	Odo-ori	12 (57.1)	9 (42.9)	12	4.610	0.032
	Adeeke	11 (100.0)	0 (0.0)	11		
	Total	23 (67.9)	9 (32.1)	23		

Crt test	Odo-ori	20 (95.2)	1 (4.8)	21	7.758	0.005
	Adeeke	5 (45.5)	6 (54.5)	11		
	Total	25 (78.1)	7 (21.9)	32		

Indo test	Odo-ori	9 (42.9)	12 (57.1)	21	4.610	0.032
	Adeeke	0 (0.0)	11 (100.0)	11		
	Total	9 (28.1)	23 (71.9)	32		

MR test	Odo-ori	12 (57.1)	9 (42.9)	21	3.841	0.049
	Adeeke	10 (90.9)	1 (9.1)	11		
	Total	22 (68.8)	10 (31.2)	32		

VP test	Odo-ori	10 (47.6)	11 (52.4)	21	4.750	0.029
	Adeeke	1 (9.1)	10 (90.9)	11		
	Total	11 (34.4)	21 (65.6)	32		

Lactose test	Odo-ori	18 (85.7)	3 (14.3)	21	0.178	0.673
	Adeeke	10 (90.9)	1 (9.1)	11		
	Total	28 (87.5)	4 (12.5)	32		

Lactose gas	Odo-ori	12 (57.1)	9 (42.9)	21	4.365	0.037^∗^
	Adeeke	10 (90.9)	1 (9.1)	11		
	Total	22 (87.5)	10 (31.2)	32		

Glucose	Odo-ori	16 (76.2)	5 (23.8)	21	1.561	0.212
	Adeeke	11 (100.0)	0 (0.0)	11		
	Total	27 (84.4)	5 (15.6)	32		

Glucose gas	Odo-ori	15 (71.4)	6 (28.6)	21	2.22	0.136
	Adeeke	11 (100.0)	0 (0.0)	11		
	Total	26 (92.9)	6 (18.8)	32		

Sucrose	Odo-ori	18 (85.7)	3 (14.3)	21	0.178	0.673
	Adeeke	10 (90.9)	1 (9.1)	11		
	Total	28 (87.5)	4 (12.5)	32		

Sucrose gas	Odo-ori	7 (33.3)	14 (66.7)	21	7.437	0.006
	Adeeke	10 (90.9)	1 (9.1)	11		
	Total	17 (53.1)	15 (46.9)	32		

## Data Availability

The data used to support the findings of this study are included within the article.
